# Switchable giant nonreciprocal frequency shift of propagating spin waves in synthetic antiferromagnets

**DOI:** 10.1126/sciadv.aaz6931

**Published:** 2020-04-24

**Authors:** Mio Ishibashi, Yoichi Shiota, Tian Li, Shinsaku Funada, Takahiro Moriyama, Teruo Ono

**Affiliations:** Institute for Chemical Research, Kyoto University, Uji, Kyoto 611-0011, Japan.

## Abstract

The nonreciprocity of propagating spin waves, i.e., the difference in amplitude and/or frequency depending on the propagation direction, is essential for the realization of spin wave–based logic circuits. However, the nonreciprocal frequency shifts demonstrated so far are not large enough for applications because they originate from interfacial effects. In addition, switching of the spin wave nonreciprocity in the electrical way remains a challenging issue. Here, we show a switchable giant nonreciprocal frequency shift of propagating spin waves in interlayer exchange–coupled synthetic antiferromagnets. The observed frequency shift is attributed to large asymmetric spin wave dispersion caused by a mutual dipolar interaction between two magnetic layers. Furthermore, we find that the sign of the frequency shift depends on relative configuration of two magnetizations, based on which we demonstrate an electrical switching of the nonreciprocity. Our findings provide a route for switchable and highly nonreciprocal spin wave–based applications.

## Teaser

We report the giant nonreciprocity and the electrical switching of propagating spin waves in synthetic antiferromagnets.

## INTRODUCTION

Nonreciprocal spin wave propagation is of great interest in the emerging research field of magnonics (*1*–*3*). This specific property provides an advantage for the enhancement of logic circuits and communication devices (*4*). It is known that the amplitude nonreciprocity in magnetostatic surface waves is caused either by the local concentration of the spin waves at the upper and lower surfaces of the ferromagnetic films (*5*) or by the nonreciprocal coupling between microwave fields and spin waves (*6*–*8*). Another nonreciprocity is the frequency shift of the propagating spin waves due to the asymmetric spin wave dispersion. This effect can be evoked by an adjacent aluminum ground plane at one end of the ferromagnetic layer due to an additional boundary condition on the tangential electric field (*9*), the difference of the surface magnetic anisotropies at the two ferromagnetic surfaces (*10*, *11*), or electrical current flows in the ferromagnets (*12*, *13*). Recent reports have shown that the nonreciprocal frequency shifts in artificial structures are attributed to the presence of an interfacial Dzyaloshinsky-Moriya interaction (i-DMI) (*14*–*19*). Although it is crucial to obtain high nonreciprocity for practical applications in spin wave logic devices, the nonreciprocal frequency shifts in the above demonstrations are limited to small values owing to interfacial effects. In addition to abovementioned interfacial effect, noncentrosymmetric magnets are one of the platforms to observe the nonreciprocal frequency shifts (*20*). Nevertheless, there are currently no solutions to satisfy practical requirements. In addition, switching of the spin wave nonreciprocity using electricity remains a challenging issue.

Here, we experimentally demonstrated a switchable giant nonreciprocal frequency shift of propagating spin waves in interlayer exchange–coupled synthetic antiferromagnets (SAFs) by spin wave spectroscopy using a vector network analyzer (VNA). The spin wave dispersion in SAFs has been first calculated and observed in the pioneering works by Grünberg *et al*. (*21*, *22*). Nonreciprocal frequency shift for thermally excited “incoherent” spin waves due to asymmetric spin wave dispersion have been experimentally observed by Brillouin light scattering techniques (*22*–*27*). In the past few years, spin wave nonreciprocity in SAFs was micromagnetically calculated and experimentally observed (*28*–*31*). However, despite the recent extensive studies on the research field of magnonics and antiferromagnetic spintronics, there have been few studies on antenna-excited “coherent” propagating spin waves in SAFs. Toward practical applications, it is necessary to investigate the characteristics of coherent propagating spin waves rather than incoherent spin waves. Moreover, switchable and highly nonreciprocal propagating spin waves will be useful for future spin wave–based applications.

### Spin wave dispersion in SAFs

**Fig. 1:**
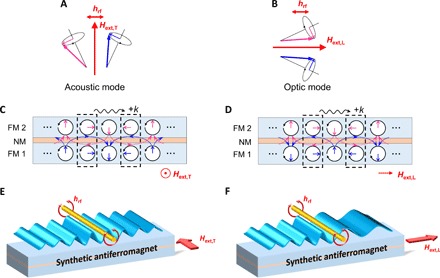
Schematic illustration of propagating spin waves in SAFs. Precessional motion of magnetizations with acoustic mode in the transverse pumping configuration (**A**) and optic mode in the longitudinal pumping configuration (**B**). The microwave field to excite the spin wave is represented by *h*_rf_. (**C** and **D**) Sketches of propagating A-SWs and O-SWs in the SAFs, which consist of two ferromagnetic layers (FM 1 and FM 2) separated by a thin nonmagnetic layer (NM). Solid blue and magenta arrows indicate the dynamic components of the magnetizations as a snapshot in time, and dotted arrows indicate the dipolar fields across the nonmagnetic layer. Spin wave dispersion becomes symmetric (C) or asymmetric (D) because of the relation between the dynamic components of the magnetization and the dipolar field as indicated by the squares in dashed line. (**E** and **F**) Conceptual illustration of this study. The wavelength of A-SWs is symmetric with respect to the propagation direction, while that of O-SWs is asymmetric.

Antiferromagnetically coupled ferromagnets exhibit two kinds of resonance precession modes: acoustic modes (in-phase precession) and optic modes (out-of-phase precession) (*22*, *32*). Considering the case of the canted magnetization state in SAFs, acoustic mode spin waves (A-SWs) can be excited when the microwave field is applied in a direction perpendicular to the bias magnetic field, namely, in a transverse pumping configuration (Fig. 1A), while optic mode spin waves (O-SWs) can be excited when the microwave field is applied along the bias magnetic field, namely, in a longitudinal pumping configuration (Fig. 1B) (*33*). The out-of-plane component of the microwave field effectively excites the A-SWs regardless of the bias magnetic field direction.

Figure 1 (C and D) illustrates the sketches of A-SWs and O-SWs in canted magnetization states under the transverse and longitudinal pumping configurations. To describe the spin wave dispersion, it is important to consider the contribution to the spin wave energy from dipolar fields generated by the magnetization motion of spin waves (*34*, *35*). Since the dipolar fields from the two ferromagnetic layers across the nonmagnetic layer are in antiphase and are canceled out in the case of A-SWs (indicated by the squares in dashed line in Fig. 1C), spin wave dispersion of A-SWs in the transverse pumping configuration is symmetric with respect to the propagation direction. Conversely, that dipolar fields are in the same phase in the case of O-SWs (indicated by the squares in dashed line in Fig. 1D). They are antiparallel (parallel) to the local magnetic moments of the two ferromagnetic layers in the forward- (reverse-) propagation direction. Therefore, spin wave dispersion of O-SWs in the longitudinal pumping configuration is asymmetric with respect to the propagation direction.

For a system including two identical ferromagnetic layers (*t*_1_ = *t*_2_ = *t* and *M*_1_ = *M*_2_ = *M*_s_), where *t* and *M*_s_ are the thickness and saturation magnetization of each ferromagnetic layer, coupled with each other through the interlayer exchange energy *J*_ex_, the resonant frequencies of A-SWs in the transverse pumping configuration *f*_T,A_ and O-SWs in the longitudinal pumping configuration *f*_L,O_ are expressed as follows (see section S2 for the derivation)

$$f_{T,A}=\frac{\mu_{0}\gamma }{2\pi }\sqrt{H_{1}H_{2}+M_{s}^{2}\frac{1-e^{-2|k|t}}{2}{\text{cos}}^{2}\phi_{0}}$$ (1)

$$f_{L,O}=\frac{\mu_{0}\gamma }{2\pi }(\sqrt{H_{1}H_{2}+H_{2}M_{s}\frac{1-e^{-2|k|t}}{2}{\text{sin}}^{2}\phi_{0}}+\text{sgn}(k)M_{s}\frac{1-e^{-2|k|t}}{4}\text{sin}\phi_{0})$$ (2)

with *H*_1_ = (*H*_ext_ cos ϕ_0_ − *H*_E_ cos 2ϕ_0_) ± *H*_E_ cos 2ϕ_0_ and *H*_2_ = (*H*_ext_ cos ϕ_0_ − *H*_E_ cos 2ϕ_0_ + *M*_s_) ± *H*_E_. The upper (lower) sign of “±” is for the resonant frequency of A-SWs (O-SWs). γ is the gyromagnetic ratio, *H*_ext_ is the external magnetic field, and *k* is the wave number. The two magnetizations become the canted state with the angle ϕ_1_ = −ϕ_2_ = ϕ_0_ = cos^−1^(*H*_ext_/2*H*_E_) in the low magnetic field region below the saturation field 2*H*_E_ = −2*J*_ex_/(*tM*_s_). According to Eq. 1, *f*_T,A_ is more dispersive as ϕ_0_ approaches zero and corresponds to the spin wave dispersion of the magnetostatic surface wave mode on a ferromagnetic film with a thickness of 2*t* above the saturation field. Conversely, according to Eq. 2, *f*_L,O_ is strongly dependent on *k* for ϕ_0_ = π/2 and no *k* dependence for ϕ_0_ = 0. Most interestingly, *f*_L,O_ differs depending on the propagating direction [*f*_L,O_(*k*) ≠ *f*_L,O_(−*k*)] in the *H*_ext_ < 2*H_E_* limit, which results in the nonreciprocal frequency shifts of the propagating spin waves. On the basis of the above discussion, the wavelength of propagating spin waves should be symmetric for A-SWs in the transverse configuration or asymmetric for O-SWs in the longitudinal configuration, as illustrated in Fig. 1 (E and F).

## RESULTS

### Sample description

**Fig. 2:**
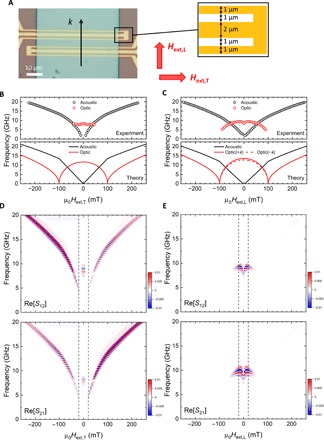
Device structure and spin wave spectroscopy measurements. (**A**) Optical micrograph of the device. The antennas in the shape of shortened coplanar waveguides are designed with a signal line (width = 2 μm) and ground lines (widths = 1 μm). (**B** and **C**) Experimentally obtained resonance peak frequencies in the spectra of Re[*S*_11_] (upper) and theoretically calculated resonance frequencies (lower) as a function of *H*_ext,T_ and *H*_ext,L_ for the acoustic mode (black symbols and lines) and the optic mode (red symbols and lines). (**D** and **E**) Two-dimensional map of transmitted signals Re[*S*_12_] and Re[*S*_21_] as a function of *H*_ext,T_ and *H*_ext,L_.

To investigate the nonreciprocal frequency shift in SAFs, we fabricated multilayers, which consisted of Ta (3 nm)/Ru (3 nm)/FeCoB (15 nm)/Ru (0.6 nm) / FeCoB (15 nm)/Ru (3 nm) on thermally oxidized Si substrates by dc magnetron sputtering. From a magnetic hysteresis loop at room temperature, the canted magnetization states of the two ferromagnetic layers were confirmed in the low magnetic field region below the saturation field of approximately 100 mT (see section S1). The films were microfabricated into devices for spin wave spectroscopy measurements (*36*), as shown in Fig. 2A (see Materials and Methods for details). We measured the scattering parameters *S*_11_, *S*_12_, *S*_21_, and *S*_22_ using a VNA at room temperature (see Materials and Methods for details). A static magnetic field was applied in the transverse direction *H*_ext,T_ and in the longitudinal direction *H*_ext,L_. Figure 2 (B and C) shows the applied magnetic field dependence of the peak frequencies in measured Re[*S*_11_] spectra (upper) and the calculated frequencies (lower) using analytical expressions of spin wave dispersion in SAFs (eqs. S8 and S9 with γ = 1.89 × 10^11^ rad/Ts, *M*_s_ = 1.21 × 10^6^ A/m, and *J*_ex_ = −9.1 × 10^−4^ J/m^2^), which yield a good agreement with the experimental data for the resonance for both the acoustic and optic modes. Note that we cannot distinguish the resonance peaks for +*k* and −*k* in the experimentally obtained Re[*S*_11_] spectra due to broad linewidth, which originate from the intrinsic damping constant and wave number distribution in the Fourier transform of the antenna current. Figure 2 (D and E) shows two-dimensional color maps of Re[*S*_12_] and Re[*S*_21_] spectra measured in the transverse pumping and the longitudinal pumping configurations. The propagating A-SWs were observed in the wide *H*_ext,T_ range, while the propagating O-SWs were observed in the low *H*_ext,L_ region below 100 mT. This can be understood from the spin wave group velocity *V*_g_, which is given by the slope of spin wave dispersion (*V*_g_ = 2π*df*/*dk*). As expressed by Eqs. 1 and 2, *V*_g_ increases (decreases) as increasing magnetic field in the ∣*H*_ext_∣ < 2*H*_E_ limit for transverse (longitudinal) pumping configuration.

### Propagating spin wave spectroscopy

**Fig. 3:**
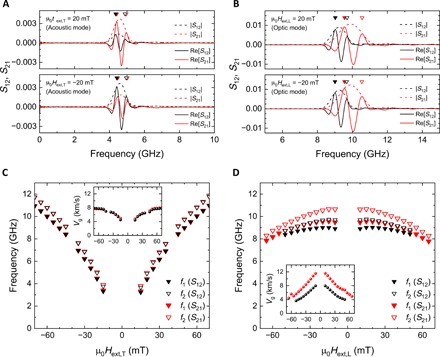
Nonreciprocal frequency shift. (**A** and **B**) Spectra of Re[*S*_12_] and Re[*S*_21_] extracted from the dashed line in Fig. 2 (D and E) under the external magnetic field of ±20 mT. The spectra of |*S*_12_| and |*S*_21_| were also plotted by dashed lines. The *S*_12_ (black line) and *S*_21_ (red line) correspond to the spin wave propagation in the forward- and reverse-propagation directions, respectively. (**C** and **D**) Peak frequencies as a function of *H*_ext,T_ and *H*_ext,L_, where solid and open triangles, respectively, correspond to the first peaks *f*_1_ and second peaks *f*_2_ in (A) and (B). Peak frequencies between *S*_12_ and *S*_21_ are almost identical for A-SWs in the transverse configuration (C) and are clearly different for O-SWs in the longitudinal configuration (D). Insets show the group velocity as a function of *H*_ext,T_ and *H*_ext,L_, estimated from the difference between *f*_1_ and *f*_2_.

To check the nonreciprocity of propagating spin waves, we extracted the Re[*S*_21_] and Re[*S*_12_] under the bias magnetic fields of ±20 mT, as indicated by the dashed lines in Fig. 2 (D and E). Regarding the propagation of A-SWs in the transverse pumping configuration (Fig. 3A), different amplitudes were observed owing to the nonreciprocal coupling between the microwave fields and spin waves depending on the propagation direction (*6*–*8*). Conversely, regarding the propagation of O-SWs in the longitudinal pumping configuration, the same amplitudes but large nonreciprocal frequency shifts of 0.65 GHz were observed depending on the propagation direction. Note that the amplitude nonreciprocities for positive (negative) field κ_+_ (κ_−_) were evaluated to be 0.39 (0.50) in the transverse pumping configuration and 0.91 (1.1) in the longitudinal pumping configuration (see Materials and Methods for details). We plotted the peak frequencies of Re[*S*_12_] and Re[*S*_21_] as a function of *H*_ext,T_ and *H*_ext,L_ in Fig. 3 (C and D), where the solid (open) triangles correspond to the first peaks *f*_1_ (second peaks *f*_2_) in Fig. 3 (A and B). The peak structures can be observed in the real part of transmitted signal when the magnetization precession between two antennas becomes in-phase. The phase difference of spin waves between *f*_1_ and *f*_2_ is 2π. In these measurements, we measured spin wave spectra by sweeping the magnetic field from positive to negative. For this sequence, the magnetization configuration always transforms from fig. S6A to fig. S6B or from fig. S6C to fig. S6D (see section S4). Therefore, the sign of nonreciprocity is not changed when the applied magnetic field direction is reversed. The insets of Fig. 3 (C and D) show the spin wave group velocities *V*_g_ estimated from (*f*_2_ − *f*_1_) ∙ *d*, where *d* (= 10 μm) is the distance between the two antennas. A large difference in *V*_g_ is observed in the propagation of O-SWs in the longitudinal pumping configuration depending on the propagation direction, which decreases as *H*_ext,L_ increases. This is consistent with the theoretical spin wave dispersion expressed by Eq. 2, whereby the nonreciprocal frequency shift is proportional to sinϕ_0_. We have also confirmed that the linear increase of the nonreciprocal frequency shift depending on *k* (see section S5). Therefore, we conclude that the nonreciprocal frequency shift in our experiment originated from mutual dipolar interaction in SAFs.

### Quantitative comparison with i-DMI

**Fig. 4:**
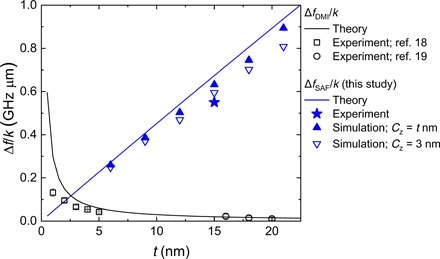
Comparison of the nonreciprocal frequency shift. The nonreciprocal frequency shift divided by the wave number Δ*f*/*k* as a function of the ferromagnetic layer thickness. The i-DMI contribution in the artificial structure (Δ*f*_DMI_/*k*, black line) and dipolar contribution in SAFs (Δ*f*_SAF_/*k*, blue line) are calculated from 2γ*D*_s_/(π*M*_s_*t*) and Eq. 2, respectively, with γ = 1.89 × 10^11^ rad/Ts, *D*_s_ = 3 pJ/m, *M*_s_ = 1.21 × 10^6^ A/m, and sin ϕ_0_ = 1. Previously reported results in Pt/Co/AlO*_x_* measured by Brillouin light scattering (squares) and Pt/Co/MgO measured by propagating spin wave spectroscopy (circles) are shown. A star symbol represents the experimentally obtained value in this study for μ_0_*H*_ext,L_ = 10 mT. The filled and open triangle correspond to Δ*f*_SAF_/*k* calculated by micromagnetic simulation with *C*_z_ = *t* nm and *C*_z_ = 3 nm, respectively, where *C*_z_ is the cell size of *z* direction.

Here, we discuss the magnitude of the nonreciprocal frequency shift of the propagating spin waves. The nonreciprocal frequency shift induced by the interfacial effect, such as i-DMI (*17*–*19*), is inversely proportional to the thickness of the ferromagnetic layer. Since *V*_g_ is proportional to the thickness of the ferromagnetic layer for the spin waves in the magnetostatic limit, it is difficult to observe the nonreciprocal frequency shift in the propagating spin wave spectroscopy. Contrary to the interfacial effect, the nonreciprocal frequency shift in SAFs is proportional to the thickness of the ferromagnetic layer owing to dipolar contributions, as expressed by Eq. 2. Figure 4 shows the systematic comparison of the nonreciprocal frequency shift as a function of ferromagnetic layer thickness between the two contributions, where Δ*f*/*k* is evaluated because the magnitude of both Δ*f*_SAF_ and Δ*f*_DMI_ is proportional to *k* in the magnetostatic limit. In the case of SAFs, we also plotted the thickness dependence of the nonreciprocal frequency shift obtained from numerical calculation with *C*_z_ = *t* nm or *C*_z_ = 3 nm, where *C*_z_ is the cell size of *z* direction (see section S3 for details). It should be noted that the nonreciprocal frequency shifts were observed in both cases although those for *C*_z_ = 3 nm are slightly smaller than those for *C*_z_ = *t* nm because of the localization of spin wave in the thickness direction depending on the propagation direction (*5*). In addition, a discretization of *C*_z_ results in the different magnetization state in the thickness direction, such as twisting of magnetization, which is not considered in the theoretically calculated spin wave dispersion and micromagnetic simulation with *C*_z_ = *t* nm. Therefore, the nonreciprocal frequency shift calculated with a discretization of *C*_z_ agrees well with the experiment. It is obvious that the dipolar contribution in SAFs reported in this study represents a larger nonreciprocal frequency shift compared to the i-DMI contribution in artificial structures. In addition, although the strength of the interlayer exchange coupling does not affect Δ*f*_SAF_ value in small magnetic field region (see section S6), SAFs with thicker ferromagnetic layers provide a further enhancement of nonreciprocal frequency shift as well as the spin wave group velocity, which will be useful for future spin wave–based applications.

### Electrically switchable nonreciprocal devices

**Fig. 5:**
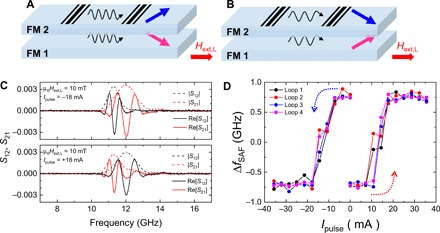
Sign reversal of nonreciprocal frequency shift based on the utilization of the current-induced Oersted field. (**A** and **B**) Two kinds of canted magnetization sates. (**C**) Propagating spin wave spectra of |*S*_12_|, |*S*_21_|, Re[*S*_12_], and Re[*S*_21_] under the external magnetic of 10 mT after applying a current pulse of −18 mA (upper) and +18 mA (lower) with 100-μs duration. (**D**) The nonreciprocal frequency shift Δ*f*_SAF_ = *f*(*S*_12_) − *f*(*S*_21_) after applying the positive and negative current pulse of various amplitudes with 100-μs duration. The switching polarity is represented by dotted arrows.

The sign of the nonreciprocal frequency shift depends on the relative magnetization angle between two ferromagnetic layers (see section S4). In the latter part of this study, we show an electrical switching of nonreciprocal frequency shift in SAFs to provide a previously unrealized manipulation technique of the relative magnetization configuration in two ferromagnetic layers (see Materials and Methods for sample details). The electrical switching of the two canted magnetization states was demonstrated as follows. First, μ_0_*H*_ext,L_ = 10 mT was applied to obtain the canted magnetizations state in SAFs. We then applied the electric current pulse *I*_pulse_ along the wire length direction with 100-μs duration, which exerts a torque on the two magnetizations in opposite directions due to current-induced Oersted field. Then, propagating spin wave spectroscopy measurements were performed after applying the current pulse of various amplitudes. Depending on the current polarity, we achieve two different canted magnetization states (Fig. 5, A and B) and succeed to switch the sign of the nonreciprocal frequency shift, as shown in Fig. 5C. To investigate the threshold current, we measured nonreciprocal frequency shift, Δ*f*_SAF_ = *f*(*S*_12_) − *f*(*S*_21_), after applying the positive and negative current pulse of various amplitudes, as shown in Fig. 5D (see section S7 for the propagating spin wave spectra as a function of *I*_pulse_). The switching of the nonreciprocal frequency shift was observed above ±18 mA corresponding to the current density of ±9.1 × 10^9^ A/m^2^. Therefore, the electrical switching of nonreciprocal frequency shift without changing the bias magnetic field can be achieved by only applying the electrical current pulse to SAFs.

## DISCUSSION

We have experimentally demonstrated the switchable giant nonreciprocal frequency shift in interlayer exchange–coupled SAFs. The magnitude of the nonreciprocal frequency shift is proportional to the thickness of the ferromagnetic layer owing to dipolar contributions between two ferromagnetic layers. It should also be noted that the wavelength symmetry of the propagating spin waves can be changed depending on the propagation direction with respect to the external magnetic field (Fig. 1, E and F), which cannot be achieved in the magnetostatic surface wave using a single ferromagnetic layer. Last, we demonstrate an electrical way to control the sign of the nonreciprocal frequency shift. Our results offer a new key for switchable and highly nonreciprocal spin wave–based applications.

## MATERIALS AND METHODS

### Sample preparation

Ta (3 nm)/Ru (3 nm)/Fe_60_Co_20_B_20_ (15 nm)/Ru (0.6 nm) / Fe_60_Co_20_B_20_ (15 nm)/Ru (3 nm) were deposited on Si/SiO_2_ substrates by dc magnetron sputtering. The two in-plane magnetized FeCoB layers separated by a Ru layer with a thickness of 0.6 nm were antiferromagnetically coupled via interlayer exchange coupling. The films were patterned into 50 μm by 100 μm wires by electron beam (EB) lithography and Ar ion milling. Subsequently, an insulating SiO_2_ layer with a thickness of 80 nm was deposited by radio frequency magnetron sputtering. Two coplanar waveguides (CPWs), which consisted of Cr (5 nm)/Au (100 nm), were then fabricated at the distance of 10 μm with the use of EB lithography and an evaporator. The designed widths of a center strip and the two side strips were 2 and 1 μm, respectively. From the calculation of the spatial distribution of the microwave current in CPWs (*36*), the spin wave with the wave number *k* of 1.2 μm^−1^ was efficiently excited in our devices.

Samples for electrically switchable nonreciprocity devices consist of Ta (3 nm)/Ru (3 nm)/Fe_40_Co_40_B_20_ (15 nm)/Ru (0.5 nm) / Fe_40_Co_40_B_20_ (15 nm)/Ru (3 nm) deposited on a Si/SiO_2_ substrate by dc magnetron sputtering. The films were patterned into 30 μm by 120 μm wires, and two CPWs and dc pads connecting to the wire to apply a dc current were fabricated by the same process as above.

### VNA measurement

Spin wave spectroscopy was performed using a VNA. The microwave probes were connected to the VNA via coaxial cables. After setting the parameters in VNA, such as the frequency range (0.01 to 20 GHz with a step of 0.01 GHz), the microwave power (−20 dBm), and bandwidth (1 kHz), the microwave apparatus was calibrated using a calibration substrate, which include short-open-load-through coplanar standards. The scattering parameters *S*_11,_ *S*_21_, *S*_12_, and *S*_22_ were measured by VNA in transverse and longitudinal pumping configurations at room temperature. Each spectrum was subtracted by a reference spectrum in the cases at which the resonant peaks were shifted away from the relevant frequency regime. On the basis of the self-scattering parameters *S*_11_ and *S*_22_, we can extract the local spin wave resonance under CPWs. On the basis of the mutual-scattering parameters *S*_12_ and *S*_21_, we can extract the propagation characteristics of the spin waves between the two CPWs.

### Amplitude nonreciprocity

Because *S* parameters has the following relation, *S* ∝ *i*2π*f*Δ*L*, where *f* is the excitation frequency and Δ*L* is a complex inductance induced by the magnetization precession, the amplitude nonreciprocity for positive field κ_+_ and negative field κ_−_ can be defined as

$$\kappa_{+}=\frac{\Delta S_{12}(f_{12})\times f_{21}}{\Delta S_{21}(f_{21})\times f_{12}}\;\text{and}\;\kappa_{-}=\frac{\Delta S_{21}(f_{21})\times f_{12}}{\Delta S_{12}(f_{12})\times f_{21}}$$

where Δ*S_ij_*(*f_ij_*) (*i*,*j* = 1,2) is the maximum spin wave amplitude at spin wave resonance frequency *f_ij_*.

## Supplementary Material

aaz6931_SM.pdf

## SUPPLEMENTARY MATERIALS

Supplementary material for this article is available at http://advances.sciencemag.org/cgi/content/full/6/17/eaaz6931/DC1
