# Supplementary material for: Switchable giant nonreciprocal frequency shift of propagating spin waves in synthetic antiferromagnets
Source: Sci Adv. 2020 Apr 24;6(17):eaaz6931. doi: 10.1126/sciadv.aaz6931 (PMC7182415; doi:10.1126/sciadv.aaz6931)
Supplement: aaz6931_SM.pdf [file aaz6931_SM.pdf]

Science Advances
AAAS

advances.sciencemag.org/cgi/content/full/6/17/eaaz6931/DC1

Supplementary Materials for

Switchable giant nonreciprocal frequency shift of propagating spin waves in synthetic antiferromagnets

Mio Ishibashi, Yoichi Shiota*, Tian Li, Shinsaku Funada, Takahiro Moriyama, Teruo Ono*

*Corresponding author. Email: shiota-y@scl.kyoto-u.ac.jp (Y.S.); ono@scl.kyoto-u.ac.jp (T.O.)

Published 24 April 2020, *Sci. Adv.* **6**, eaaz6931 (2020)
DOI: 10.1126/sciadv.aaz6931

This PDF file includes:

Sections S1 to S7
Figs. S1 to S9

SUPPLEMENTARY MATERIALS

S1 *M-H* loop

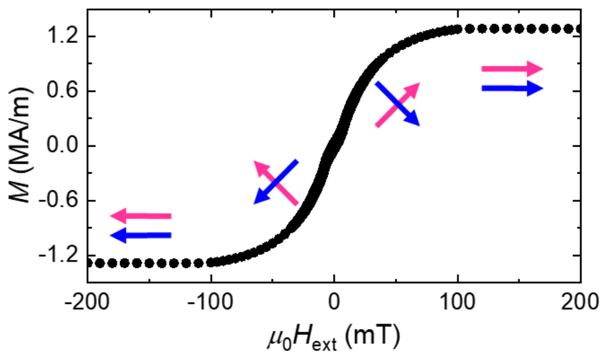

Fig. S1 *M-H* loop of FeCoB/Ru/FeCoB synthetic antiferromagnet under in-plane magnetic field.

Figure S1 shows a *M-H* loop of FeCoB(15 nm)/Ru(0.6 nm)/FeCoB(15 nm) synthetic antiferromagnet under in-plane magnetic field. Both FeCoB layers were antiferromagnetically aligned at zero magnetic field due to the interlayer exchange-coupling. The canted magnetization state of two layers was confirmed in low magnetic region below the saturation field of approximately 100 mT.

S2 Analytical expression of spin wave dispersion in synthetic antiferromagnets

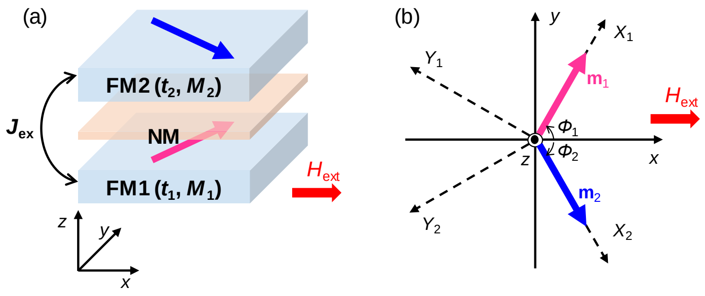

Fig. S2 (a) Schematic view of the film structure. (b) Coordinate system considered in the calculation.

The system including two identical ferromagnetic layer (FM1 and FM2; $t_1 = t_2 = t$, $M_1 = M_2 = M_s$), where t and M_s are the thickness and the saturation magnetization of each FM layer, and the interlayer exchange energy per unit surface area J_{ex} through the nonmagnetic (NM) layer is considered as shown in Fig. S2(a). The coordinate system considered in this study is illustrated in Fig.

S2(b). The unit vectors pointing in the magnetization direction of FM1 and FM2 are denoted as $\mathbf{m}_1 = (m_{x1}, m_{y1}, m_{z1})$ and $\mathbf{m}_2 = (m_{x2}, m_{y2}, m_{z2})$. The external magnetic field H_{ext} lies in the x direction. The magnetic energy density E of this system can be described by

$$E = -tM_s H_{\rm ext}(m_{x1} + m_{x2}) + \frac{1}{2} tM_s^2 (m_{z1}^2 + m_{z2}^2) + J_{\rm ex}\mathbf{m}_1 \cdot \mathbf{m}_2. \qquad \text{(S1)}$$

The first term in Eq. (S1) is the Zeeman energy, the second term is the demagnetization energy, and the last term is the interlayer exchange energy. The equilibrium magnetization angle ϕ_0 is determined from the condition $(\partial E/\partial \phi)_{\theta=\frac{\pi}{2}} = 0$,

$$\phi_1 = -\phi_2 = \phi_0 = \begin{cases} \cos^{-1}\frac{H_{\rm ext}}{2H_{\rm E}} & \left(H_{\rm ext} < 2H_{\rm E} = -2\frac{J_{\rm ex}}{tM_s}\right) \\ 0 & (H_{\rm ext} \geq 2H_{\rm E}) \end{cases}. \qquad \text{(S2)}$$

Eq. (S2) indicates that two magnetizations are canted when the external magnetic field is smaller than the saturation field $2H_E$.

The magnetization dynamics are described by the following Landau-Lifshitz equation

$$\frac{d\mathbf{m}_i}{dt} = -\gamma \mathbf{m}_i \times \mathbf{H}_i \quad (i = 1,2) \qquad \text{(S3)}$$

where γ is the gyromagnetic ratio, and $\mathbf{H}_i$ is the effective magnetic field. In the presence of spin wave, $\mathbf{H}_i$ can be expressed as

$$\mathbf{H}_i = H_{\rm ext}\mathbf{e}_x - M_s m_{zi}\mathbf{e}_z - H_{\rm E}\mathbf{m}_j - \mathbf{H}_{\rm dip,i} \quad (i, j = 1,2 \text{ and } i \neq j). \qquad \text{(S4)}$$

Here, $\mathbf{H}_{dip,i}$ denotes the dipolar field generated by the magnetization motion of spin waves. From the Maxwell's relations $\nabla \cdot \mathbf{B} = 0$ and $\nabla \times \mathbf{H}_{\rm dip} = 0$, $\mathbf{H}_{dip,i}$ is expressed as follows(35).

$$\mathbf{H}_{\rm dip,i} = M_s \frac{1-e^{-2|k|t}}{4}\left[\begin{pmatrix} -\cos^2\phi_k & 0 & 0 \\ 0 & -\sin^2\phi_k & 0 \\ 0 & 0 & 1 \end{pmatrix}\delta\mathbf{m}_i + \right.$$
$$\left.\begin{pmatrix} -\cos^2\phi_k & -\sin\phi_k\cos\phi_k & -i\cos\phi_k \\ -\sin\phi_k\cos\phi_k & -\sin^2\phi_k & -i\sin\phi_k \\ -i\cos\phi_k & -i\sin\phi_k & 1 \end{pmatrix}\delta\mathbf{m}_j\right]. \qquad \text{(S5)}$$

where k is the wavenumber, ϕ_k is the angle between the external magnetic field and spin wave propagation direction, and $\delta\mathbf{m}$ is the component of the magnetization precession. The first and

second term in Eq. (S5) express the self-dipolar field and the mutual-dipolar field, respectively. ϕ_k = 0, π and ϕ_k = π/2, 3π/2 correspond to the longitudinal pumping configuration and the transverse pumping configuration.

Now we introduce a new $X_iY_iZ_i$-coordinate where X_i axis is parallel to the magnetization in the equilibrium state, as shown in Fig. S2(b). The transformation from the *xyz*-coordinate to the $X_1Y_1Z_1$-coordinate is performed by multiplying the following rotation matrix to Eq. (S3):

$$\hat{\mathbf{R}}_1 = \begin{pmatrix} \cos\phi_0 & \sin\phi_0 & 0 \\ -\sin\phi & \cos\phi & 0 \\ 0 & 0 & 1 \end{pmatrix}. \tag{S6}$$

A similar expression can be obtained on $\mathbf{m}_2$. Considering a small oscillation of the magnetization around the equilibrium state with the approximations $m_{Xi} \simeq 1$, and $|m_{Yi}|,|m_{Zi}| << 1$, Eq. (S3) is linearized as

$$i2\pi f \begin{pmatrix} m_{Y1} \\ m_{Z1} \\ m_{Y2} \\ m_{Z2} \end{pmatrix} = -\gamma \hat{H} \begin{pmatrix} m_{Y1} \\ m_{Z1} \\ m_{Y2} \\ m_{Z2} \end{pmatrix}, \tag{S7}$$

The explicit form of a 4th order matrix $\hat{H}$ depends on the direction of spin wave propagation. The resonant frequency f is eigenvalue of the matrix $\hat{H}$. It is known that the antiferromagnetically coupled ferromagnetic layers show two kinds of resonance mode. One is the in-phase precession known as the acoustic mode and the other is the out-of-phase precession known as the optic mode. Using the expression for $\mathbf{H}_i$ in Eq. (S4) and (S5), the spin wave frequency of acoustic mode f_A and optic mode f_O can be obtained by solving Eq. (S7) for each configuration.

$$\text{Longitudinal: } \begin{cases} f_{L,A} = \frac{\mu_0\gamma}{2\pi}\left(\sqrt{H_1H_2 - H_1M\frac{1-e^{-2|k|t}}{2}} - \mathrm{sgn}(k)M\frac{1-e^{-2|k|t}}{4}\sin\phi_0\right) \\ f_{L,O} = \frac{\mu_0\gamma}{2\pi}\left(\sqrt{H_1H_2 + H_2M\frac{1-e^{-2|k|t}}{2}\sin^2\phi_0} + \mathrm{sgn}(k)M\frac{1-e^{-2|k|t}}{4}\sin\phi_0\right) \end{cases} \tag{S8}$$

$$\text{Transverse: } \begin{cases} f_{T,A} = \frac{\mu_0\gamma}{2\pi}\sqrt{H_1H_2 + M^2\frac{1-e^{-2|k|t}}{2}\cos^2\phi_0} \\ f_{T,O} = \frac{\mu_0\gamma}{2\pi}\sqrt{H_1H_2} \end{cases} \tag{S9}$$

where $H_1 = (H_{ext}\cos\phi - H_E\cos 2\phi) \pm H_E\cos 2\phi$ and $H_2 = (H_{ext}\cos\phi - H_E\cos 2\phi + M_s) \pm$

H_E. The double sign "±" means the upper (+) for the acoustic mode and the lower (-) for the optic mode.

In $H_{ext} \geq 2H_E$ limit ($\phi_0 = 0$), the spin wave dispersions in acoustic mode ($f_{L,A}$ and $f_{T,A}$) correspond to that for the magnetostatic backward volume wave (MSBVW) mode and magnetostatic surface wave (MSSW) mode on a ferromagnetic film thickness of $2t$, whereas the spin wave dispersions in optic mode ($f_{L,O}$ and $f_{T,O}$) is independent of k, which means no spin wave propagation. On the other hand, in $H_{ext} < 2H_E$ limit ($\phi_0 = \cos^{-1}\frac{H_{ext}}{2H_E}$), the ϕ_0 dependent terms in Eq. (S8) and (S9) become important. Especially, the spin wave dispersions in the longitudinal configuration ($f_{L,A}$ and $f_{L,O}$) depend on the sign of k. This nonreciprocity [$f(k) \neq f(-k)$] is caused by the symmetry of the dipolar field expressed by the second term of Eq. (S5).

S3 Numerical results and discussion

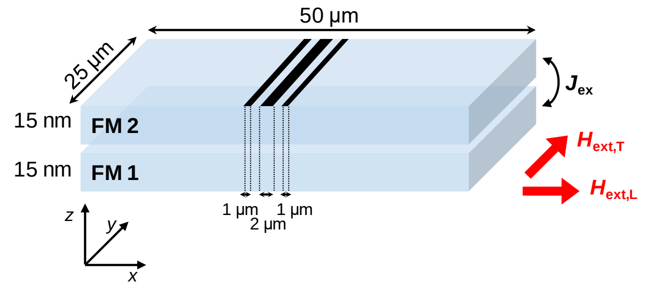

Fig. S3 Schematic structure used in the micromagnetic simulation. The rf magnetic field is applied at the antennas, colored with black at the center of synthetic antiferromagnet.

To test the validity of analytical expression of spin wave dispersions, we performed micromagnetic simulation using MuMax3 code. The material parameters of FeCoB used in the simulations are the damping constant $\alpha = 0.01$, the gyromagnetic ratio $\gamma = 1.89 \times 10^{11}$ rad/Ts, the saturation magnetization $M_s = 1.21 \times 10^6$ A/m, and the exchange constant $A = 1.0 \times 10^{-11}$ J/m. As shown in Fig. S3, the length of the thin film is 50 μm in the x direction, the width is 25 μm in the y direction, and the thickness of each FM layer is 15 nm in the z direction. We used $50 \times 50 \times C_z$ nm^3

mesh, where C_z is the cell size of z direction. To verify the effect of spin wave localization in the z direction, we chose C_z = 15 nm (1 cell per CoFeB layer) and C_z = 3 nm (5 cell per CoFeB layer), which is smaller than the exchange length $l_{ex} = \sqrt{2A/\mu_0 M_s^2} \cong 3.3$ nm. NM spacer layer is assumed to be negligibly thin, but we rescale the exchange coupling in the region between two ferromagnetic layers by the interlayer exchange energy J_{ex} = -9.1 × 10^{-4} J/m^2 to obtain antiferromagnetic coupling. The damping at the ends of the waveguide is assumed to be 1 to suppress the spin wave reflection. To excite spin waves, we apply an rf magnetic field ($h_{rf}\sin(2\pi ft)$, 0, 0) in a center strip with 2-μm-width and ($-h_{rf}\sin(2\pi ft)$, 0, 0) in two side strips with 1-μm-width, where $\mu_0 h_{rf}$ = 1 mT. It should be noted that the antenna design does not affect the spin wave dispersion. The results were recorded at $50/f$ s after spin wave excitation.

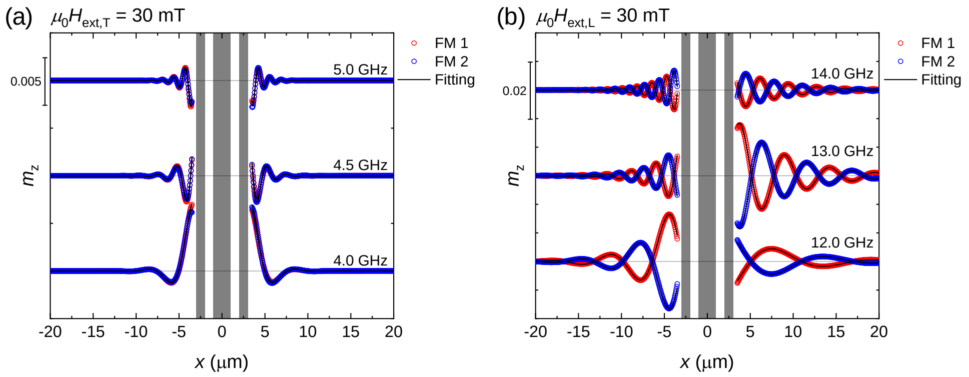

Fig. S4 (a,b) Snapshot images of m_z-component distribution in FM1 and FM2 along x axis for (a) $\mu_0 H_{ext,T}$ = 30 mT and (b) $\mu_0 H_{ext,L}$ = 30 mT, respectively.

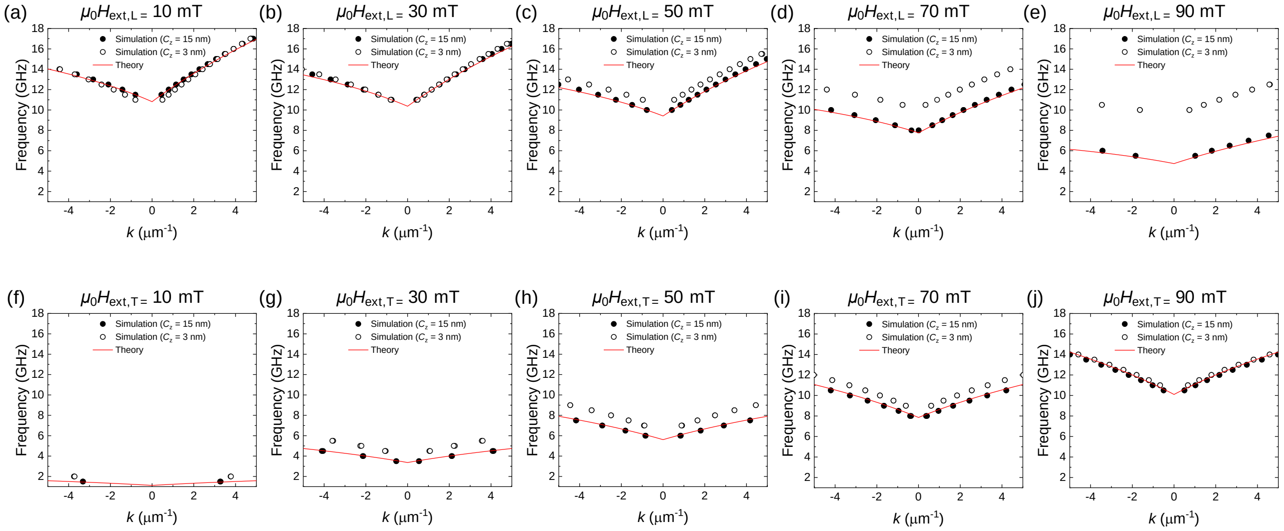

Fig. S5 Spin wave dispersions of (a-e) optic mode in the transverse pumping configuration and (f-j) acoustic mode in the longitudinal pumping configuration. The filled and open symbols correspond to numerical results with C_z = 15 nm and C_z = 3nm and red lines corresponds to analytical results.

Figures S4 (a) and (b) show snapshot images of m_z-component distribution in FM1 and FM2 along x axis for (a) $\mu_0 H_{ext,T}$ = 30 mT and (b) $\mu_0 H_{ext,L}$ = 30 mT, respectively. Propagating spin waves with acoustic mode (in-phase precession) and optic mode (out-of-phase precession) were clearly observed in transverse pumping configuration and longitudinal pumping configuration, as experimentally demonstrated in the main text. Then, these results are fitted to an exponentially decaying sine function, as shown in Figs. 4 (a) and (b). From these fits we determine the wavenumber k as a function of the frequency. Figures S5 (a-j) show the spin wave dispersions in each configuration. Numerical results with C_z = 15 nm agree well with analytical expression of spin wave dispersions [Eqs. (S8) and (S9)]. However, those with C_z = 3 nm were slightly different from analytical results. This might be due to the localization of spin wave in the thickness direction depending on the propagation direction.

S4 Magnetization alignments in longitudinal pumping configuration

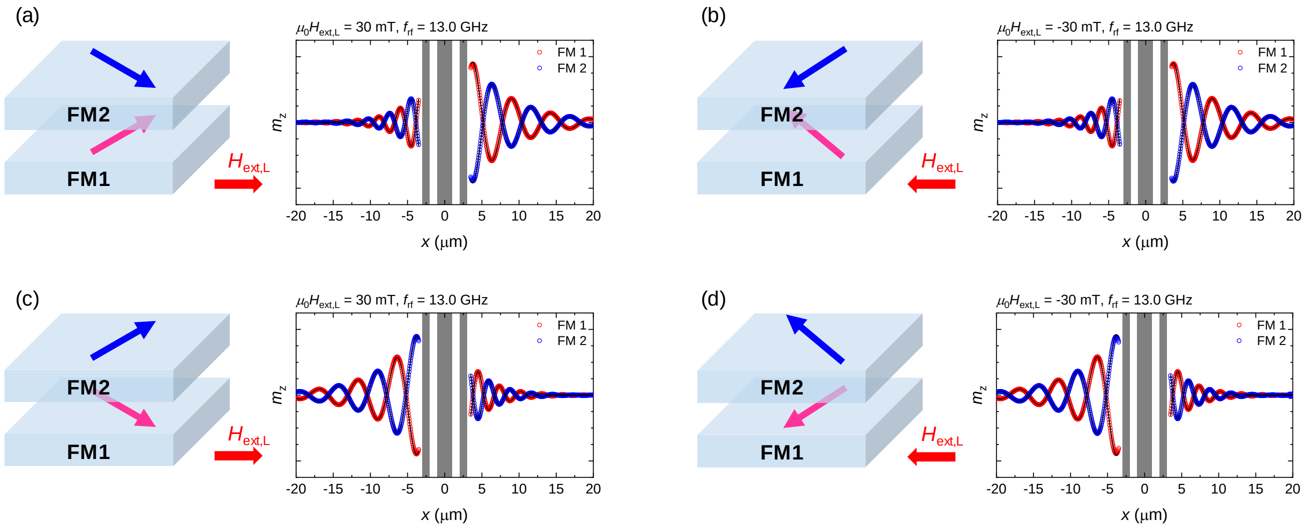

Fig. S6 (a-d) Left: magnetization alignments in two ferromagnetic layers under the corresponding magnetic field direction. Right: snapshot images of m_z-component distribution in FM1 and FM2 along x axis.

Eq. (S8) indicates that the sign of frequency shift depends on not only the propagating direction but also the sign of equilibrium magnetization angle ϕ_0. Since $\phi_1 = -\phi_2 = \phi_0$ and $\phi_1 = -\phi_2 = -\phi_0$ energetically identical, two magnetization alignments are possible under the fixed external magnetic field, as shown in Figs. S5 (a), (c) and (b), (d). From micromagnetic simulations, we confirm that the sign of frequency shift is opposite between ϕ_0 and $-\phi_0$.

S5 Dependence of the nonreciprocal frequency shift on wavenumber

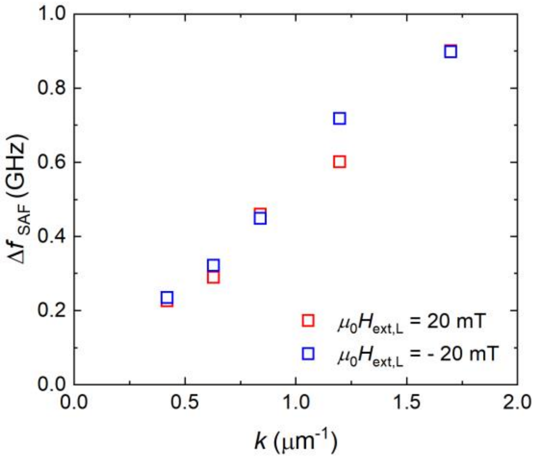

Fig. S7 Nonreciprocal frequency shift of SAFs Δf_{SAF} for $\mu_0 H_{ext,L} = \pm 20$ mT as a function of wavenumber.

To confirm the asymmetric dispersion in the present system, we investigated k-dependence of the nonreciprocal frequency shift Δf_{SAF} by changing the width of CPWs. Figure S5 shows the k-dependence of Δf_{SAF} for $\mu_0 H_{ext,L}$ = ±20 mT. The obtained Δf_{SAF} increases linearly with increasing k, which agrees with the theoretical spin wave dispersion of Eq. (S8) Therefore, we conclude that the nonreciprocal frequency shift in our experiment originated from mutual dipolar interaction in the SAF structure.

S6 Dependence of the spin wave dispersion on the interlayer exchange coupling strength

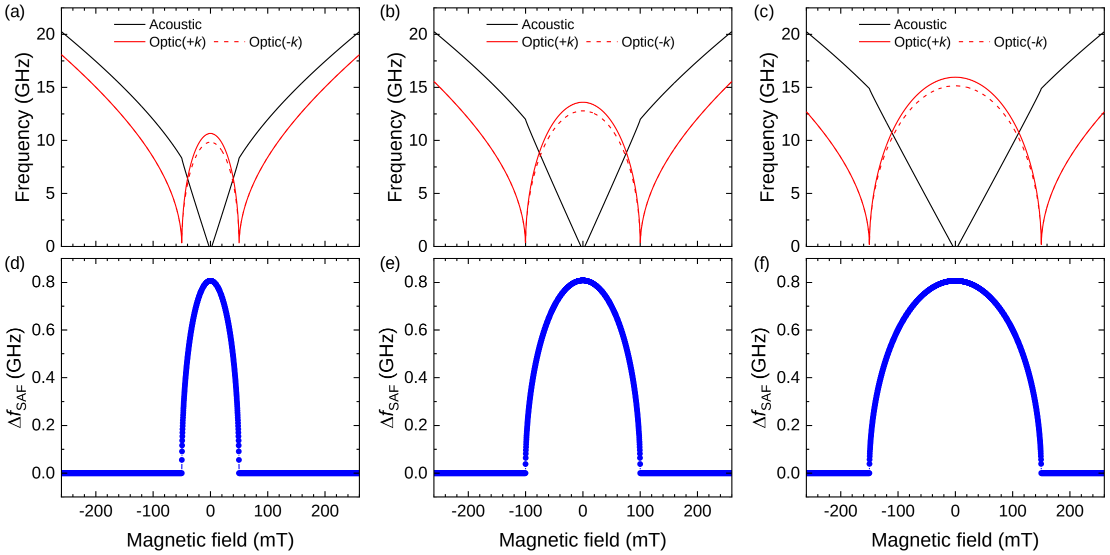

Fig. S8 (a-c) Theoretically calculated resonance frequencies as a function of $H_{ext,L}$ for the acoustic mode and the optic mode and (d-f) nonreciprocal frequency shift for the optic mode with $\mu_0 2H_E$ = 50 mT (J_{ex} = 4.5 × 10^{-4} J/m^2) [(a) and (d)], $\mu_0 2H_E$ = 100 mT (J_{ex} = 9.1 × 10^{-4} J/m^2) [(b) and (e)], and $\mu_0 2H_E$ = 150 mT (J_{ex} = 13.6 × 10^{-4} J/m^2) [(c) and (f)].

Dependence of the spin wave dispersion on the interlayer exchange coupling strength J_{ex} are theoretically calculated using Eq. (S8), as shown in Fig. S6. As increasing J_{ex}, although the resonance frequency for spin wave with optic mode becomes high in the small magnetic field region, the nonreciprocal frequency shifts at zero magnetic field are identical. This can be interpreted from Eq. (S8). The nonreciprocal frequency shift for O-SW in the longitudinal pumping configuration can be expressed as follows,

$$\Delta f_{\mathrm{SAF}} = M_s \frac{1-e^{-2|k|t}}{2} \sin \phi_0. \tag{S10}$$

Note that the J_{ex} value is included in ϕ_0, which is the equilibrium magnetization angle at canted magnetization configuration. Therefore, the strength of interlayer exchange coupling doesn't affect Δf_{SAF} value in small magnetic field region.

S7 Propagating spin wave spectra after applying the current pulse

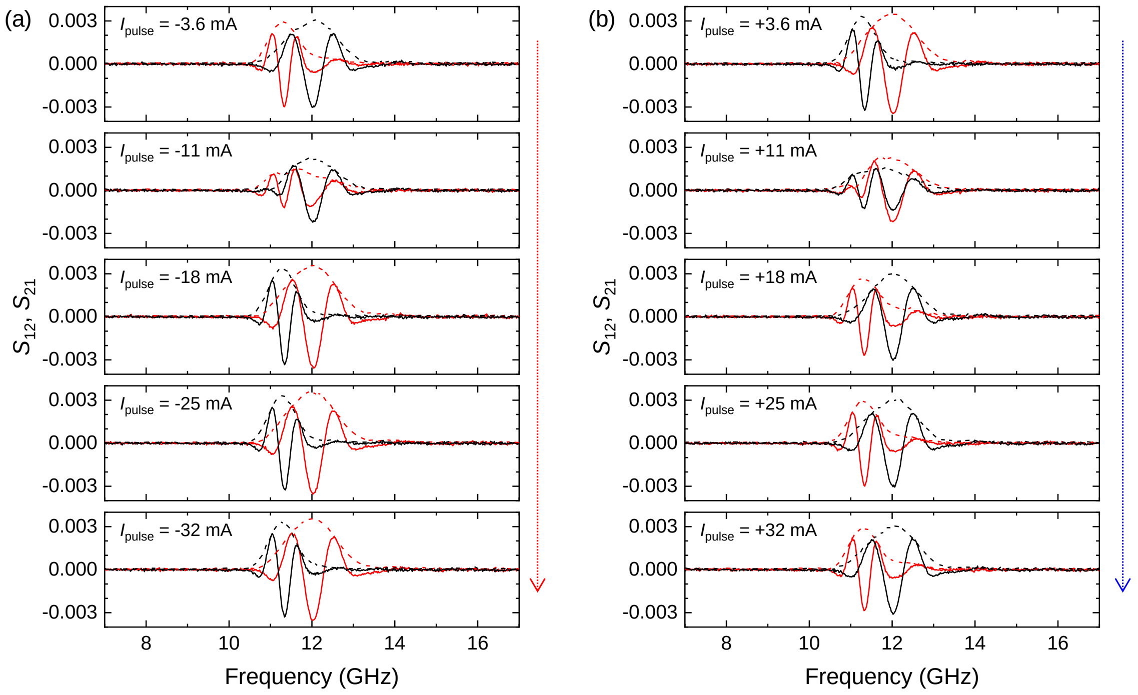

Fig. S9 Propagating spin wave spectra of $|S_{12}|$, $|S_{21}|$, Re[S_{12}] and Re[S_{21}] under $\mu_0 H_{\mathrm{ext,L}}$ = 10 mT after applying current pulse with 100 μs duration for (a) negative I_{pulse} and (b) positive I_{pulse}.
